# Exploration of the Immune-Related Long Noncoding RNA Prognostic Signature and Inflammatory Microenvironment for Cervical Cancer

**DOI:** 10.3389/fphar.2022.870221

**Published:** 2022-05-19

**Authors:** Hui Yao, Xiya Jiang, Hengtao Fu, Yinting Yang, Qinqin Jin, Weiyu Zhang, Wujun Cao, Wei Gao, Senlin Wang, Yuting Zhu, Jie Ying, Lu Tian, Guo Chen, Zhuting Tong, Jian Qi, Shuguang Zhou

**Affiliations:** ^1^ Department of Gynecology, Anhui Medical University Affiliated Maternity and Child Healthcare Hospital, Hefei, China; ^2^ Department of Gynecology, Anhui Province Maternity and Child Healthcare Hospital, Hefei, China; ^3^ Department of Pharmacy, North China University of Science and Technology, Tangshan, China; ^4^ Department of Clinical Laboratory, Anhui Medical University Affiliated Maternity and Child Healthcare Hospital, Anhui Province Maternity and Child Healthcare Hospital, Hefei, China; ^5^ Department of Radiation Oncology, the First Affiliated Hospital of Anhui Medical University, Hefei, China; ^6^ Anhui Province Key Laboratory of Medical Physics and Technology, Center of Medical Physics and Technology, Hefei Institutes of Physical Science, Chinese Academy of Sciences, Hefei, China

**Keywords:** TCGA, immune-related, inflammation, lncRNA, cervical cancer, prognosis, risk score, tumor inflammatory microenvironment

## Abstract

**Purpose:** Our research developed immune-related long noncoding RNAs (lncRNAs) for risk stratification in cervical cancer (CC) and explored factors of prognosis, inflammatory microenvironment infiltrates, and chemotherapeutic therapies.

**Methods:** The RNA-seq data and clinical information of CC were collected from the TCGA TARGET GTEx database and the TCGA database. lncRNAs and immune-related signatures were obtained from the GENCODE database and the ImPort database, respectively. We screened out immune-related lncRNA signatures through univariate Cox, LASSO, and multivariate Cox regression methods. We established an immune-related risk model of hub immune-related lncRNAs to evaluate whether the risk score was an independent prognostic predictor. The xCell and CIBERSORTx algorithms were employed to appraise the value of risk scores which are in competition with tumor-infiltrating immune cell abundances. The estimation of tumor immunotherapy response through the TIDE algorithm and prediction of innovative recommended medications on the target to immune-related risk model were also performed on the basis of the IC50 predictor.

**Results:** We successfully established six immune-related lncRNAs (AC006126.4, EGFR-AS1, RP4-647J21.1, LINC00925, EMX2OS, and BZRAP1-AS1) to carry out prognostic prediction of CC. The immune-related risk model was constructed in which we observed that high-risk groups were strongly linked with poor survival outcomes. Risk scores varied with clinicopathological parameters and the tumor stage and were an independent hazard factor that affect prognosis of CC. The xCell algorithm revealed that hub immune-related signatures were relevant to immune cells, especially mast cells, DCs, megakaryocytes, memory B cells, NK cells, and Th1 cells. The CIBERSORTx algorithm revealed an inflammatory microenvironment where naive B cells (*p* < 0.01), activated dendritic cells (*p* < 0.05), activated mast cells (*p* < 0.0001), CD8^+^ T cells (*p* < 0.001), and regulatory T cells (*p* < 0.01) were significantly lower in the high-risk group, while macrophages M0 (*p* < 0.001), macrophages M2 (*p* < 0.05), resting mast cells (*p* < 0.0001), and neutrophils (*p* < 0.01) were highly conferred. The result of TIDE indicated that the number of immunotherapy responders in the low-risk group (124/137) increased significantly (*p* = 0.00000022) compared to the high-risk group (94/137), suggesting that the immunotherapy response of CC patients was completely negatively correlated with the risk scores. Last, we compared differential IC50 predictive values in high- and low-risk groups, and 12 compounds were identified as future treatments for CC patients.

**Conclusion:** In this study, six immune-related lncRNAs were suggested to predict the outcome of CC, which is beneficial to the formulation of immunotherapy.

## Introduction

Cervical cancer (CC), a type of gynecological malignancy, is a serious threat to women’s health. It currently ranks as the fourth highest incidence and mortality rate of gynecologic cancers around the world (Bray et al., 2018). Mortality rates in poorly paid countries and territories are 18 times higher than in developed countries, which varies widely, with 85% of fatality occurring in less developed countries because of the restricted therapeutic schedule and socioeconomic and cultural conditions (Small et al., 2017). Currently, routine treatments for CC include surgery, chemotherapy, and radiation, but terminal cancer patients are suitable to develop radiation and chemical treatment tolerance (Seol et al., 2014). In addition, within 2 years of treatment, most CC patients have a poor prognosis due to the recurrence or metastasis of the tumor (Liontos et al., 2019). Thus, it is very important to find novel prognostic targets and therapeutic strategies to enhance the viability of CC patients.

Inflammation is an antidamage reaction to endogenous or exogenous injury in the body, and tumor-associated inflammation is incredibly important in tumor progression. It has been found that a large number of inflammatory cells are found in tumor tissues that transmit considerable amounts of cytokines, chemokines, tumor necrosis factor-α, and macrophage migration inhibitory factors (Greten and Grivennikov, 2019).

These persistent anti-infective factors can exacerbate DNA damage in cells, inducing cell mutations that lead to cell proliferation. Tumor cells convert some of the inflammatory substances into transmitters that favor the tumor growth and extension as the tumor progresses. Tumor cells can release cytokines and chemokines to appeal to immune cells to promote cancer progression. The immune system is able to identify tumor-associated surface antigens and activate cells to produce a series of immune responses to attack and eliminate tumor cells, which ultimately leads to the release of various effect molecules which can stop tumors from growing (Barabas et al., 2018). The disequilibrium of the tumor inflammatory microenvironment (TIME) has vital implications for the genesis and evolution of tumors. By now, immunotherapy has become an innovative therapy for CC in the process of cellular molecular biology and immunology (Ventriglia et al., 2017). The mechanism of tumor immunotherapy is mainly to provoke and strengthen the antitumor immune response by increasing the immunogenicity of tumor cells and the sensitivity of cell damage, and the other is that the immune cells and molecules that work in the body prompt the immune system not only to kill smaller cancer cells but also to suppress the recurrence and metastasis of the tumor (O'Donnell et al., 2019).

Long noncoding RNAs (lncRNAs) are transcripts of more than 200 nucleotides, which are regions of the genome that cannot encode proteins. More evidence indicates that lncRNAs generally decontrol in cervical malignancy. lncRNAs play an important role on a variety of biological functions, for instance, gene transcription, translation, and modification, through acting upon proteins, RNA, and DNA. In recent decades, there have been studies showing that lncRNAs are involved in tumorigenesis of CC. For example, the downregulation of a long noncoded RNA called lnc-CCDST in CC tissue promoted the vitality and angiogenesis of tumor cells by being bound to cancer-causing DHX9 that upregulated in the CC tissue (Ding et al., 2019). Another study demonstrated the clinical prognostic importance of the lncRNA SOX21-AS1 in hypomethylation for CC (Wang et al., 2019a). lncRNA BBOX1 antisense RNA 1 induced the growth and proliferation of cancer cells by a higher HOXC6 expression by way of miR-361-3p and HuR (Xu et al., 2020). Therefore, lncRNAs could be implicated as potential biomarkers in the therapy and prognosis of CC. Nevertheless, it is a pity that the mechanisms of immune-related lncRNAs in the prognosis of CC have not been clearly explained yet. We carried out this study for the purpose of identifying immune-related RNAs, which might act as a prognostic and therapeutic target for CC. The survival analysis, Cox regression risk model, and other analytical methods were utilized to exploit prognostic signatures and pathogenesis of immune-related lncRNAs. Obviously, it is necessary for us to illuminate hub lncRNAs in cervical cancer that will forcefully impact the future execution and management of therapeutic strategies for patients.

## Materials and Methods

### Data Acquisition

The transcriptome profile data of CC were collected from TCGA TARGET GTEx datasets in UCSC Xena (https://xena.ucsc.edu/), including 306 tumor samples and 13 matched normal samples. The normalized gene expression was measured as transcripts per million (TPM) and log2-based transformation. In addition, integral survival information and the clinical materials of 274 CC patients were obtained from TCGA database, covering age, TNM stages, tumor grades, and vital status at tracking patients and so on, aiming to analyze the survival prognosis of differentially expressed lncRNAs (DELncRNAs). Simultaneously, we sorted out a list of lncRNA signatures from the GENCODE database (https://www.gencodegenes.org/) so that DELncRNAs were analyzed significantly. In addition, we acquired an immune-related signature list from the Immunology Database and Analysis Portal (ImmPort) database (https://www.immport.org/) containing 2,396 immune-related genes, which serves as a usable material for immunological research.

### Inclusion and Exclusion Criteria for Patients Used to Construct Risk Signatures

The inclusion criteria included primary tumor patients, complete clinical pathology parameters, and patients with transcriptome sequencing data. The primary outcome measure was overall survival (OS) and follow-up time ≧ 30 days. Patients whose survival status and clinical information are incomplete were excluded.

### Preliminary Data Processing

We performed the “limma” package in R to examine the normalization of expression profiling and executed analysis of difference by false discovery rate (FDR) < 0.05 and | log folding changes | > 1 to find out genes that were abnormally expressed in tumors and normal samples. We then obtained significant DELncRNAs by intersecting lncRNA signatures with differentially expressed genes (DEGs) on a Venn diagram.

### Foundation of Hub Immune-Related lncRNA Signatures and the Immune-Related Risk Model

By taking advantage of the “survival” package in R, we conducted univariate Cox regression between DELncRNAs and OS to determine survival-associated DELncRNAs. Then, survival-associated immune-related DELncRNAs with absolute values of correlation coefficient >0.6 were screened using the Pearson’s rank correlation test between survival-associated DELncRNAs and immune-related genes. Then, applying the “glmnet” package in R, we employed the least absolute shrinkage and selection operator (LASSO) regression model to minimize overfitting and determine the most remarkable survival-associated immune-related DELncRNAs in CC. After collinear testing, a step-by-step multivariate Cox regression analysis was carried out to establish the hub immune-related lncRNAs (survival-associated immune-related DELncRNAs derived) risk signature in CC. The visualization of the multivariate Cox regression analysis was demonstrated by the “forestplot” package in R. Taking the Cox coefficient and gene expression together, the risk score was calculated as this equation (k,**β**i, and Si mean the number of genes, coefficient index, and gene expression level, respectively) (Lossos et al., 2004; Chen et al., 2007; Hu et al., 2019):

$$Model:risk\;score=\sum_{i=1}^{\kappa }\beta iSi.$$

Referring to the median risk score, we classified the sample into high- and low-risk groups.

The Kaplan–Meier curve and log-rank test were then employed to assess the survival between the two groups. In addition, the risk map was described in the “ggrisk” package. To this step, the immune-related risk model was successfully constructed.

### Determination of the Factors That Effects on the Prognostic Outcome of CC

Since the identified hub immune-related lncRNAs were related to CC survival results, we planned to further study the predictive value of these signatures. To assess independent prognosis factors, we applied the “forestplot” package to evaluate four major clinical parameters including age, staging, grading, and the risk level of the immune-related risk model. Moreover, a nomogram composed of primary clinical parameters and prognostic factors was formulated through multivariate Cox regression with the application of “rms,” “foreign,” and “survival” packages.

Through calculating Harrell’s concordance index (C-index), the calibration curves of the nomogram for 1-, 3-, and 5-year OS were drawn to evaluate the performance of the prognostic nomogram in the accuracy of the actual observation rate and the probability of the predicted survival. Meanwhile, the “survival ROC” package performed the receiver operating characteristic (ROC) curves to display 1-, 3- and 5-year OS predictions to assess the prognosis veracity with the hub immune-related lncRNAs. The accuracy of 1-, 3-, and 5-year ROC curves was analyzed by decision curve analysis (DCA).

### Inference of the Correlation Between Immune-Related lncRNAs and Tumor-Infiltrating Immune Cells in xCell

With a view to measure the abundance of tumor interstitial cells in CC patients, we used a package called “xCell,” which combined the benefits of gene enrichment with deconvolution methods to fit RNA-seq sequence and microarray data and estimated the synthetic levels of 64 stromal cells based on gene signatures. According to the “xCell” package instructions, the xCell signature (*N* = 64) was allowed to run with 1000 permutations, which was eventually incorporated into 64 cell types for the study.

### Verification of the Infiltrating Cells in CIBERSORT and the Difference in Immune Cell Abundance in High- and Low-Risk Groups

Using CIBERSORTx, a deconvolution algorithm, 22 cell types, including B cells, T cells, natural killer (NK) cells, macrophages, and dendritic cells (DCs), figured out a confirmable *p*-value in each sample by Monte Carlo sampling. These cases were analyzed with *p* < 0.05 due to the high credibility of the deduced cellular constituent. The abundance variation of immune cells in high- or low-risk groups was described in the “pheatmap” package. Afterward, we used a box plot to display the results, and different immune cells were noted in the study. Next, the Wilcoxon rank-sum test was employed to contrast the difference in abundance of immune cells in two risk groups. Last, we made a Kaplan–Meier analysis with OS for 22 immune cells.

### Discovery of the Model in Immunotherapy and Potential Compounds

The tumor immune dysfunction and exclusion (TIDE) algorithm was utilized to predict the post-treatment response of immunotherapy methods. The TIDE algorithm is a computational model used to simulate two main nosogenesis of tumor immune escape (T-cell dysfunction and T-cell exclusion). TIDE signatures were effective and preferably given immunotherapy biological targets that could provide predictive immunotherapy reactions for CC. Next, we obtained the IC50 of compounds in the Genomics of Drug Sensitivity in Cancer (GDSC) website to speculate on potential biomarkers for CC therapy in clinical settings and performed a correlation and difference analysis with IC50 predictive values for 27 chemotherapeutics commonly used to treat cervical cancer (detailed information in Supplementary Table S2). The IC50 of compounds was calculated by the “pRRophetic” package in R.

### Statistical Analysis

The aforementioned statistical methods were carried out by R software (version 4.1.0). The “limma” package was applied to differential analysis and normalization. The correlation analysis used Pearson’s rank correlation test. The statistical difference in survival rates of two risk groups was assessed using the Kaplan–Meier curve and log-rank test. The prognosis influencing factors were analyzed using multivariate Cox regression analysis, the results of which are illustrated by a nomogram. The ROC curve determined the predictive accuracy of hub genes, the accuracy of which was confirmed by the DCA. The Wilcoxon rank-sum test was utilized to select differential expression genes, infiltrative immune cells, and IC50 predictive values in the different groups. *p* value <0.05 was regarded as statistically significant.

## Results

### Identification of DELncRNA Signatures in CC

**FIGURE 1:**
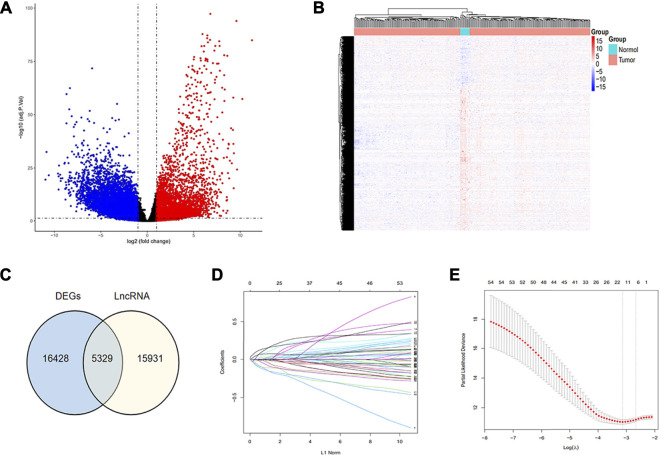
Recognition of hub immune signatures in CC. **(A,B)** Volcano plot and heat map were drawn to exhibit the difference in expression genes between tumor and normal samples; **(C)** Venn diagram was drawn to show the obtained DELncRNA signatures through intersecting lncRNA signatures with DEG; **(D,E)** Twelve hub immune-related lncRNAs were identified using the LASSO regression method.

Our study procured 319 samples from the TCGA TARGET GTEx datasets in UCSC Xena (https://xena.ucsc.edu/), consisting of 306 tumor samples versus 13 normal samples. The gene expression was normalized as transcripts per million (TPM) and log2-based transformation. Meanwhile, the corresponding survival data and clinical information of CC patients were acquired in TCGA database, and all clinical material is demonstrated in Supplementary Table S1. We excluded patients with insufficient survival information and found that 274 samples met the requirements of full survival and transcriptome data, which were brought into subsequent studies. The “limma” package was employed to pick out 16,428 differentially expressed genes (DEG) by means of |log fold change| >1 and false discovery rate (FDR) < 0.05. The result was visualized using the volcano diagram and heatmap to display up- or downregulation of the aforementioned genes (Figures 1A,B). We listed lncRNA signatures from the GENCODE database and obtained 5,329 DELncRNA signatures approximately, by intersecting lncRNA signatures with DEGs, which is exhibited in a Venn diagram (Figure 1C), consisting of 1,433 upregulated and 3,896 downregulated genes that were identified.

### Identification of Prognostic Immune-Related lncRNA Signatures in CC

First, we merged the expression data of DELncRNAs with survival information. Utilizing the “survival” package, we conducted univariate Cox regression on DELncRNAs and OS of CC in TCGA database to identify 453 survival-associated DELncRNAs (*p* < 0.05). With 2,396 immune-related genes procured from the ImmPort database, we further explored to screen the prognostic-relevant signatures among them. Then, 54 survival-associated immune-related DELncRNAs with absolute values of correlation coefficient >0.6 were screened using Pearson’s rank correlation test between survival-associated DELncRNAs and immune-related genes.

### Establishment of the Hub Immune-Related lncRNA Signature and Risk Model

**FIGURE 2:**
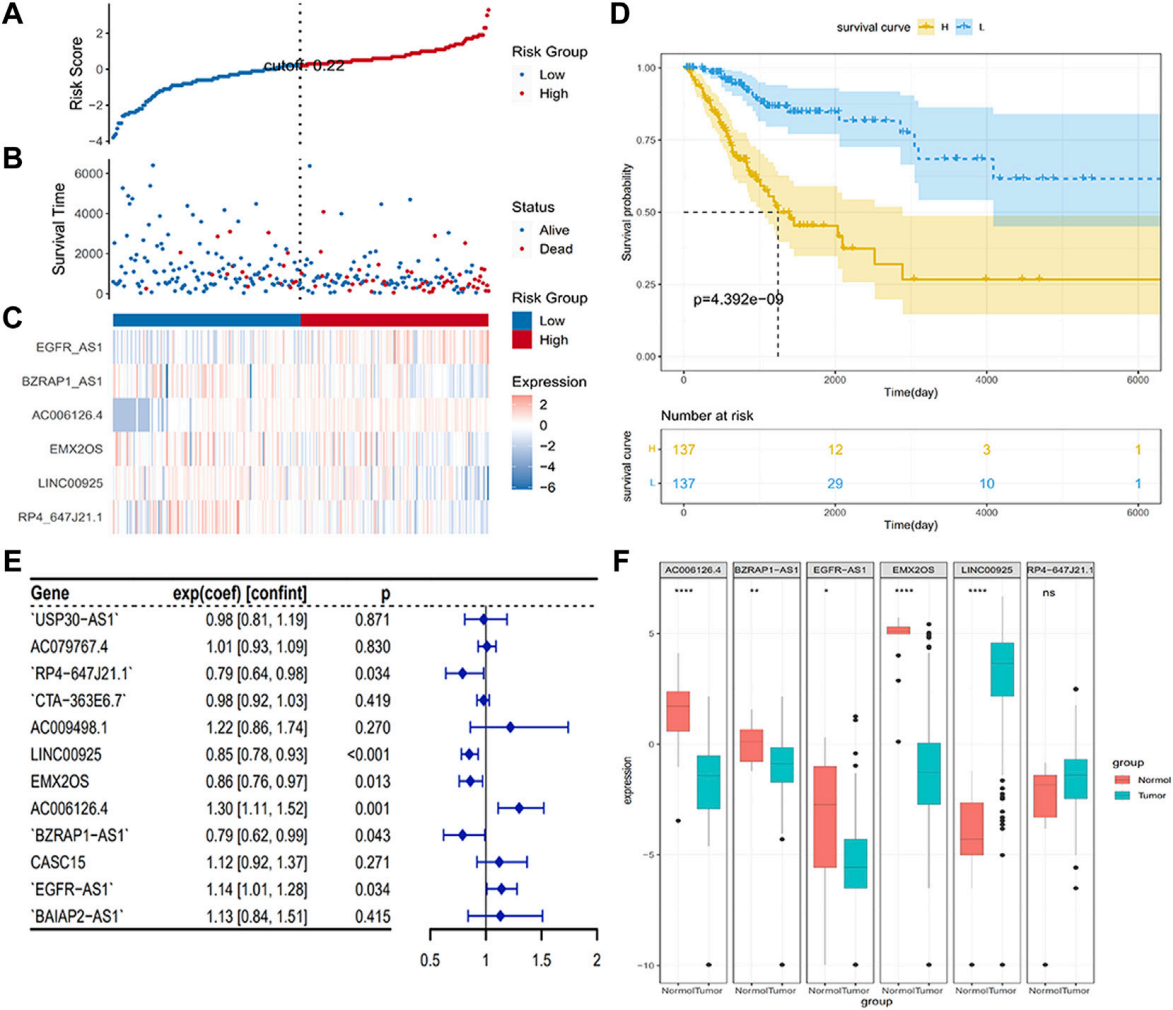
Establishment of hub genes and distributions. **(A,B)** Survival states of patients depending on hub signature levels; **(C)** Heatmap plot was used to determine variant levels in hub signatures identified between different groups; **(D)** Kaplan–Meier analysis with the hub immune signature; **(E)** Forest plot visualized results of multivariate Cox regression analysis; **(F)** Differences in expression levels of six hub genes in different groups.

Considering that these immune-related signatures exhibited different expression quantities in tumor or normal samples, we ulteriorly screened out the hub genes. We performed the LASSO regression method and multivariate Cox regression analysis to find the most significant prognostic immune-related lncRNAs. In the LASSO regression method, as the filter program showed in Figures 1D,E, we identified 12 lncRNAs. Next, we figured out each coefficient of signature through a multivariate Cox regression analysis and the result of which is demonstrated in Figure 2E. Last, six lncRNAs were identified in the hub immune-related lncRNAs. The weight of each hub gene is shown in Table 1. The risk score of hub immune-related lncRNAs was accordingly formulated as Risk Score = −0.233882 × RP4-647J21.1 −0.159569 × LINC00925 −0.154896 × EMX2OS +0.260937 × AC006126.4 −0.238834 × BZRAP1-AS1 +0.128459 × EGFR-AS1. Therefore, we established an immune-related risk model successfully that divides 274 samples into high- and low-risk groups, with 137 cases in each group. It was easy to see that patients with higher scores had a higher risk of survival, as exhibited in Figures 2A,B. In addition, the heatmap and the box plot displayed different expression levels of the hub genes in the high-risk group versus the low-risk group and the tumor group versus normal group, respectively (Figures 2C,F). The heatmap illustrated that AC006126.4 and EGFR-AS1 showed the overexpression in the high-risk group, but RP4-647J21.1, LINC00925, EMX2OS, and BZRAP1-AS1 had medium-even underexpression levels. We also used Kaplan–Meier analysis to further study hub lncRNAs, as shown in Figure 2D, to indicate that patients with higher risk scores were bound with lower OS (*p* < 0.001).

**TABLE 1:** Results of hub lncRNAs based on TCGA TARGET GTEx data after the multivariate Cox regression.

	Description	coef	exp (coef)	se (coef)	z	Pr (>|z|)
RP4-647J21.1	NA	−0.234	0.791	0.110	−2.123	0.034
LINC00925	Long intergenic non-protein coding RNA 925, also known as the MIR9-3 Host Gene	−0.160	0.853	0.046	−3.496	0.000
EMX2OS	EMX2 opposite strand/antisense RNA	−0.155	0.857	0.062	−2.487	0.013
AC006126.4	NA	0.261	1.298	0.081	3.219	0.001
BZRAP1-AS1	BZRAP1 antisense RNA 1, also known as TSPOAP1-AS1	−0.239	0.788	0.118	−2.020	0.043
EGFR-AS1	EGFR antisense RNA 1	0.129	1.137	0.061	2.120	0.034

### Establishment of Prognostic Factors in CC From TCGA

**FIGURE 3:**
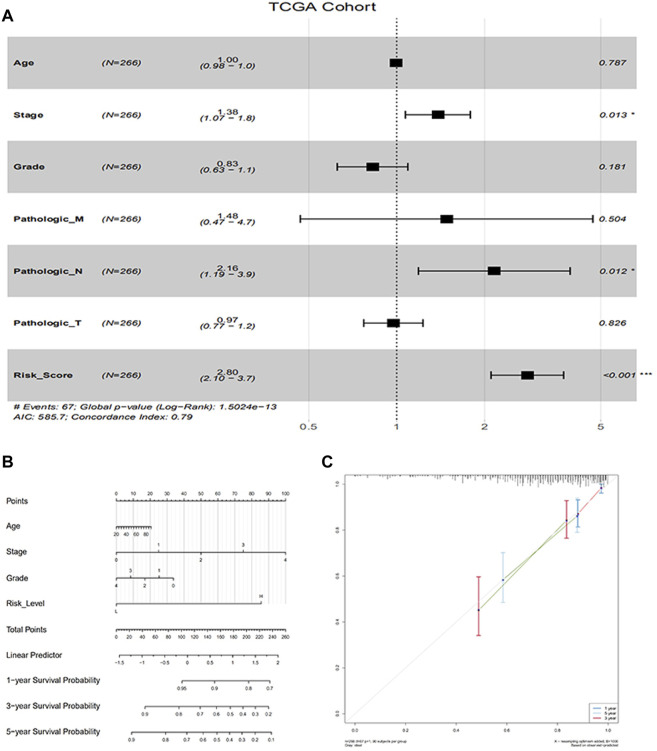
Evaluation of factors in impacting prognosis of patients for CC. **(A)** Multivariable Cox proportion hazard regression for OS of CC; **(B)** Nomogram comprised risk scores and other clinical parameters for the prediction of 1-, 3-, and 5-year OS of CC; **(C)** Calibration plot of the nomogram for probabilistic forecasts of 1-, 3-and 5-year overall survival of patients.

**FIGURE 4:**
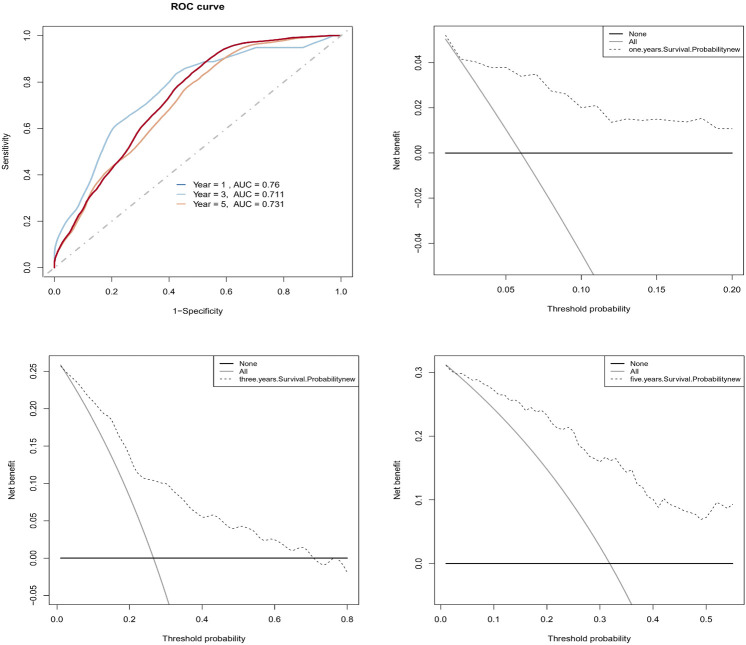
Evaluation of the predictive prognosis capability of CC by hub signatures. **(A)** The 1-, 3-, 5-year AUC of ROC curve was 0.760, 0.711, and 0.731, indicating better predictive power; **(B, C, D)** The decision curve analysis (DCA) estimated the accuracy of predicting 1-, 3-, 5-year prognosis.

To find independent prognostic factors for signatures, including age, stage, grade, pathologic M, pathologic N, pathologic T, OS, OS time, risk level, and risk score, the package of “gg forest” was applied. Results of the gg forest plot showed that stage (HR = 1.38, *p* < 0.05), pathologic N (HR = 2.16, *p* < 0.05) and risk score (HR = 2.80, *p* < 0.001) had predictive values for OS in CC, as shown in Figure 3A. Performing the “rms” package, four primary clinical variables, including age, stage, grade, and risk levels, were shown in the nomogram (Figure 3B), which also showed a prediction of 1-, 3-, or 5-year OS. At the same time, we verified that the C-index of the nomogram was 0.799 and testified a desirable consistency between the prediction and observed values at the odds of 1-, 3-, and 5-year survival (Figure 3C). The aforementioned result demonstrated that the constructed nomogram had manifested a satisfactory prediction in the survival rate of CC patients in 1, 3, or 5 years. If we refer to the earlier content, each sample was included in a high- or low-risk group. On the basis of 6 lncRNAs, we used time-dependent ROC analyses of 1, 3, and 5 years to estimate the precision of prognosis, resulting in 1-, 3-, and 5-year AUC corresponding values of 0.760, 0.711, and 0.731 (Figure 4A).

In addition, we successfully confirmed the uniformity of probabilities between the actual and predicted survival rates by DCA (Figures 4B–D).

### Associations of Immune-Related lncRNAs With Immune Cells Using xCell

**FIGURE 5:**
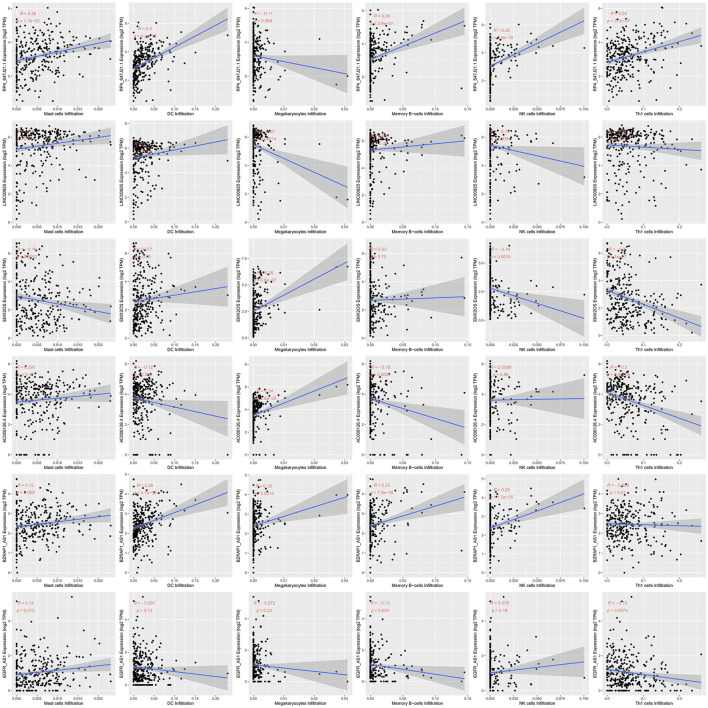
Correlations between hub signatures and tumor-infiltrating immune cells.

**FIGURE 6:**
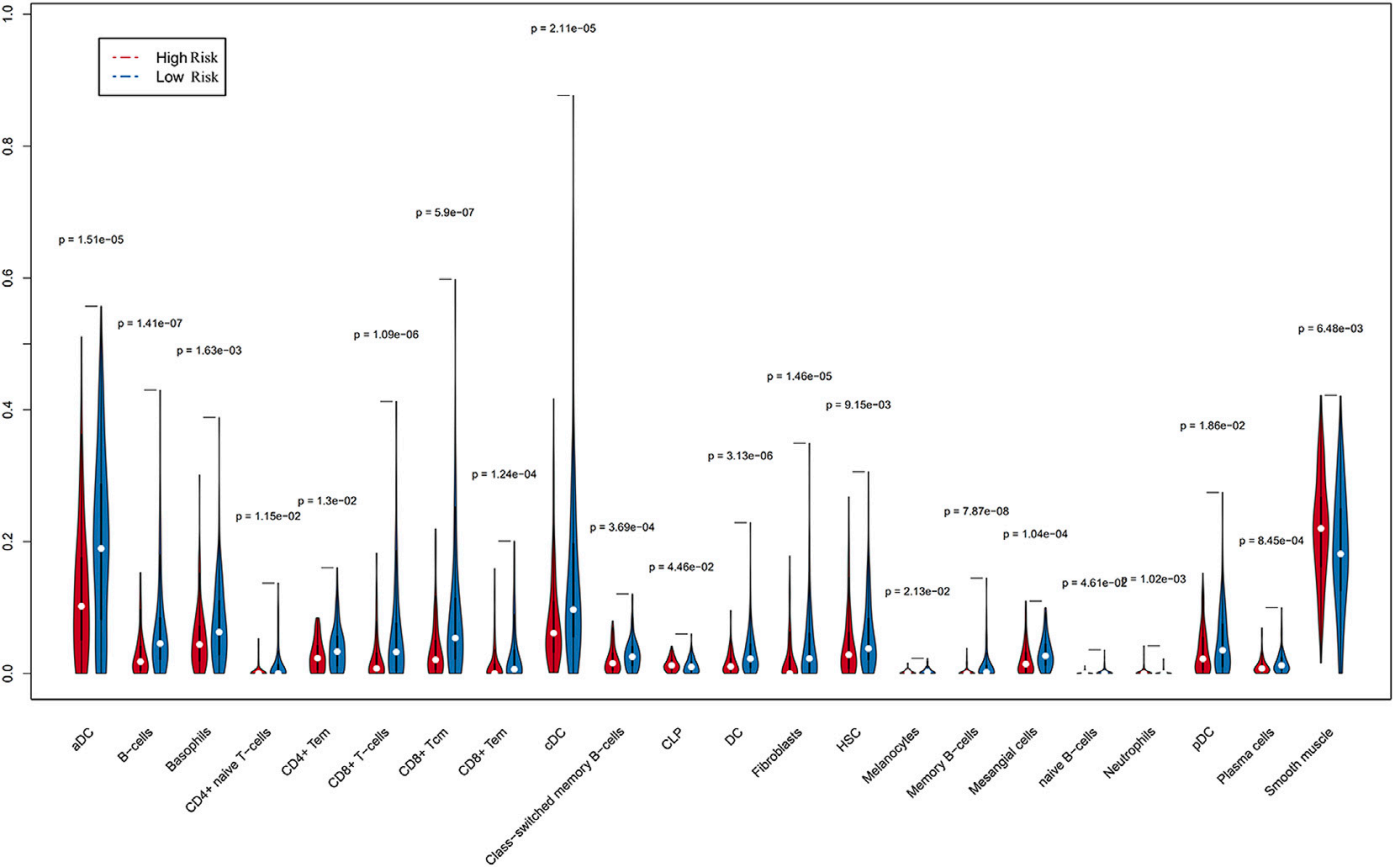
Differences in immune cells screened from 64 mesenchymal cells were accurately compared by Wilcoxon rank and testing (*p* < 0.05), indicating that a few immune cells gave different infiltrating densities in two risk groups.

Since immune cell infiltration plays a very important role in the TIME, we combined immune-related signatures with immune infiltration cells into an overall research. On basis of the xCell algorithm, most of the six hub lncRNAs were correlated with the following important immune cells, as shown in Figure 5, such as mast cells, DCs, megakaryocytes, memory B cells, NK cells, and Th1 cells. Moreover, we made use of the violin plot to demonstrate the differences of several immune cells in the TIME between the high- and low-risk groups (Figure 6). Filtering out some immune cells from 64 mesenchymal cell species, we were left with immune cells with *p* < 0.05. The reflected differences of typical immune cells with high- and low-risk groups in the TIME by using a violin diagram, such as aDCs, B cells, basophils, CD4^+^ naive T cells, CD4^+^ T effector memory (Tem) cells, CD8^+^ T cells, CD8^+^ T central memory (Tcm) cells, CD8^+^ Tem cells, cDCs, class-switched memory B cells, common lymphoid progenitor (CLP) cells, DCs, fibroblasts, hematopoietic stem cells (HSCs), melanocytes, memory B cells, mesangial cells, naive B cells, neutrophils, pDCs, plasma cells, and smooth muscle cells. Compared between immune cells, CC tissue generally contained higher proportions of aDCs, basophils, CD8^+^ Tcm cells, cDCs, and smooth muscle cells, whereas CD4^+^ naive T cells, mesangial cells, naive B cells, and neutrophil fractions were relatively lower. Overall, we found less infiltration of these immune cells in the high-risk group than in the low-risk group. In other words, the reduction of immune-infiltrating cells in the tumor microenvironment is possibly associated with poor prognosis for CC. Moreover, we concluded that the infiltration levels of CD8^+^ T cells far exceeded those of CD4^+^ cells. Similar to this report’s finding, CD8^+^ T cells were preferred to be recruited into cervical lesions and progressed to invasive (Edwards et al., 1995).

### Differential Abundance of Tumor-Infiltrating Immune Cells in High- or Low-Risk Groups Using CIBERSORT

**FIGURE 7:**
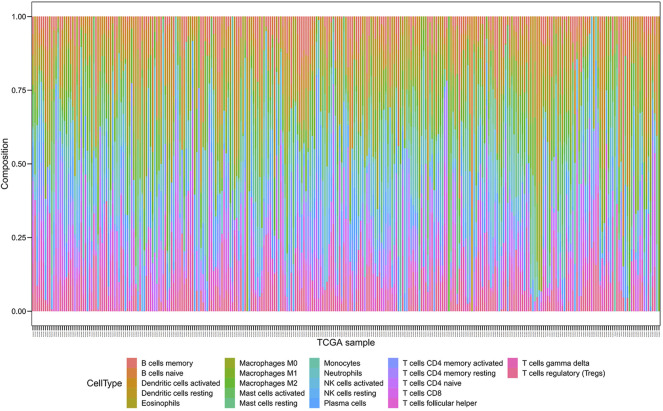
Composition of immune cells were evaluated through the CIBERSORT algorithm, and 22 immune cells were annotated with different colors in the legend.

**FIGURE 8:**
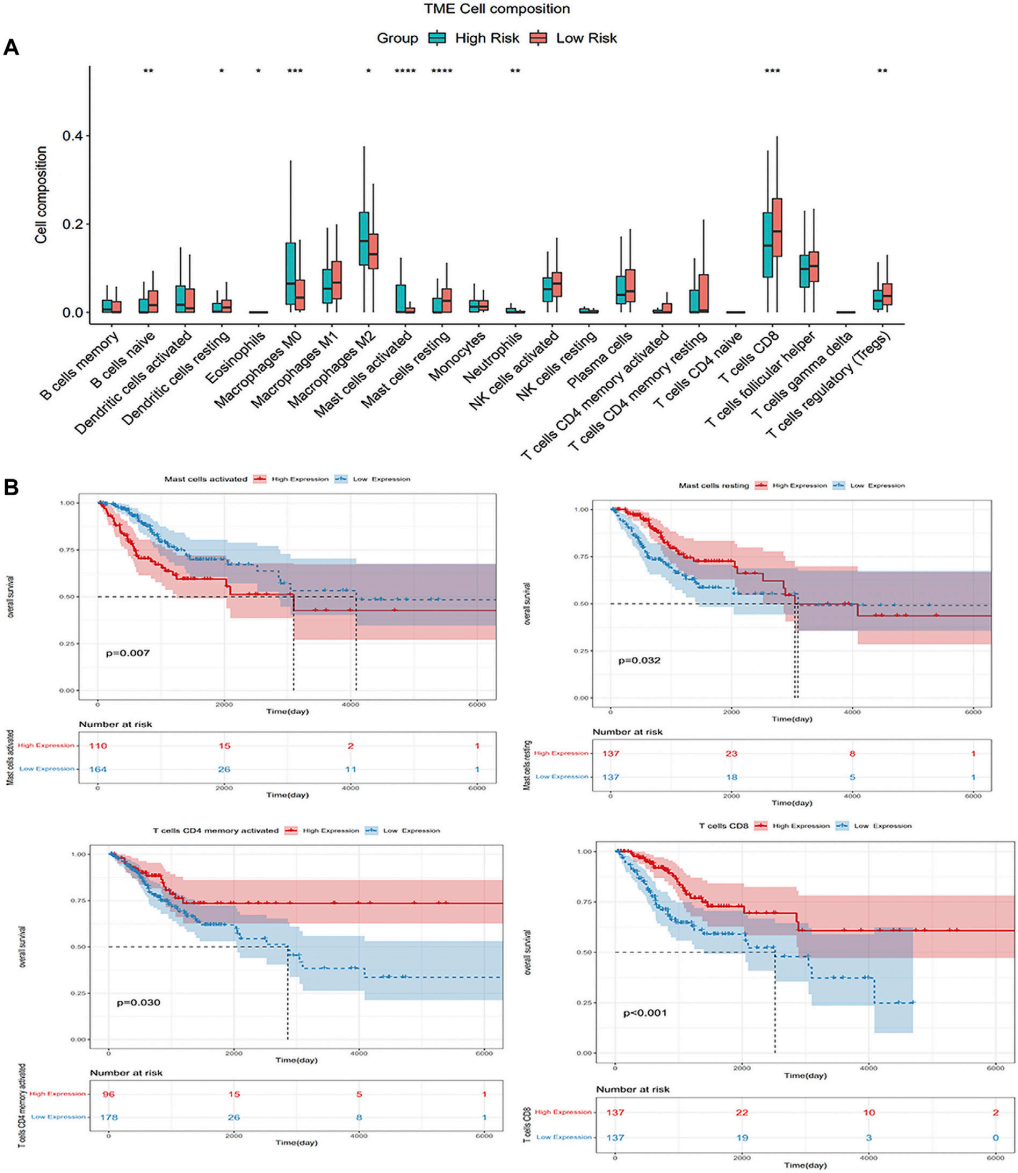
**(A)** Wilcoxon rank-sum tests compared the difference and manifested that some immune cells had different infiltrating levels in two risk groups; **(B)** Kaplan–Meier analysis with OS for 22 immune cells.

We employed the CIBERSORT algorithm to pre-estimate the infiltration level of 22 immune cells in each sample. To ensure the accuracy of the analysis, we retained a sample with a *p*-value > 0.05, and the heatmap showed the difference of immune cells in infiltration abundance between the two groups, which were annotated in different colors, with the sum of the immune fractions in each sample equal to 1 (Figure 7). The potential association between immune signatures and several immune cells was analyzed, so it is assumed that the distribution of immune cells was different in the two risk groups (Figure 8A). The Kaplan–Meier curve exhibited the difference in prognosis analyzed by high- and low-risk group immune cells together with OS and finally retains the significant immune cells of *p* < 0.05 (Figure 8B). The result was described in a box plot *via* the Wilcoxon rank-sum test to accurately contrast the difference in the two risk groups and discovered that a few immune cells conferred a noticeably lower infiltrating degree in the high-risk groups, containing naive B cells (*p* < 0.01), activated dendritic cells (*p* < 0.05), activated mast cells (*p* < 0.0001), CD8^+^ T cells (*p* < 0.001), and regulatory T cells (*p* < 0.01). Conversely, some immune cells were higher conferred in the cancer samples, such as macrophages M0 (*p* < 0.001), macrophages M2 (*p* < 0.05), resting mast cells (*p* < 0.0001), and neutrophils (*p* < 0.01). It was also found that CD8^+^ T cells and macrophages were the most inundated immune cells. We made the Kaplan–Meier analysis with OS for 22 immune cells and found that the following four immune cells are meaningful: activated mast cells (*p* = 0.007), resting mast cells (*p* = 0.032), resting memory CD4^+^ T cells (*p* = 0.030), and CD8^+^ T-cells (*p* < 0.001).

### Estimation of Cancer Immunotherapy Response and New Chemotherapy Candidates Aimed at the Immune-Related Risk Model

**FIGURE 9:**
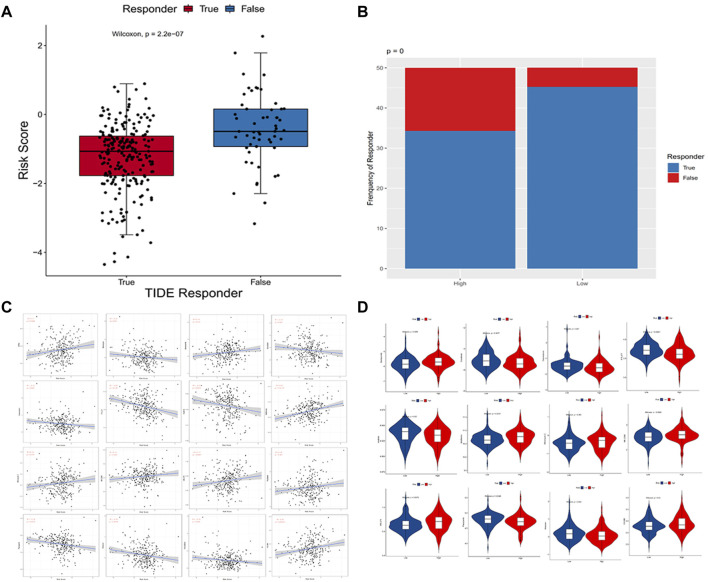
**(A,B)** Distribution of immunotherapeutic response in different groups based on the TIDE algorithm. Wilcoxon rank-sum tests were employed to analyze contingency tables for ICI responders; **(C)** Point plots exhibited the correlation between 16 compounds and the estimated IC50; **(D)** Violin plots visualized the differences in the estimated IC50 between these two groups.

Using the TIDE algorithm, the effect of immunotherapy response can be predicted by transcriptomic data so as to explore whether the immunocortical models can predict the benefits of immunotherapy in CC patients. The final table was obtained after the TIDE algorithm, as shown in Supplementary Table S3. Based on the results of the Wilcoxon test, in the immune-related risk model, low-risk patients (124/137) were significantly higher than high-risk patients (94/137) (*p* = 0.00000022) (Figure 9A), and the risk score was significantly inversely relevant to the immunotherapy response of CC patients (Figure 9B). To treat CC patients, we further speculated about potential agents targeting the immune-related risk model. The pRRophetic algorithm was utilized to assess the treatment response of each sample supported by the half-maximal inhibitory concentration (IC50) that was obtained from the Genomics of Drug Sensitivity in Cancer (GDSC) database.

We performed a correlation analysis between IC50 predictive values of 27 drugs and risk scores (Supplementary Table S2) commonly used to treat cervical cancer and found that 16 compounds were in significant correlation with the estimated IC50 (*p* < 0.05) (Figure 9C), and 12 of the 16 compounds were screened because of the significant differences in the approximated IC50 (*p* < 0.05) comparing the two groups. According to the correlation and difference analysis results of IC50 predicted values, it showed that 12 compounds (bortezomib, docetaxel, doxorubicin, FTI.277, imatinib, metformin, mitomycin.C, MK.2206, MS.275, pazopanib, shikonin, and VX.680) could be employed to further study targeting CC patients. At the same time, high-risk scores were correlated with a lower IC50 of these chemotherapy drugs and were found to be doxorubicin (*p* = 0.025), FTI.277 (*p* = 0.000004), imatinib (*p* = 0.0037), pazopanib (*p* = 0.0015), and shikonin (*p* = 0.0016). Conversely, it was related to a higher IC50 of these drugs, such as bortezomib (*p* = 0.012), docetaxel (*p* = 0.047), metformin (*p* = 0.00000022), mitomycin.C (*p* = 0.019), MK.2206 (*p* = 0.032), MS.275 (*p* = 0.0057), and VX.680 (*p* = 0.00025), which demonstrated these compounds as possible potential indicators of treatment for chemical sensitivity (Figure 9D).

## Discussion

Over the past couple of years, inflammatory and immune diseases have become a global focus of health concern. Immunity is a kind of defense function of the human body. It not only relies on this function to identify “self” and “non-self” components, destroy, and reject antigenic substances entering the body but also monitor and identify and remove abnormal cells produced in the body at any time to prevent the production of tumor cells. Inflammation and immunity are intimately concerned, and more evidence suggests that the host’s inflammatory response can be contributed significantly in the development and progression of cancer by influencing the immune microenvironment, such as in CC, inflammation is a significant agent affecting the disease to develop, progress, and thus leading to metastasis. Hence, better understanding of the common pathways between inflammation and cancer and investigating the alteration of the TIME may pave the way for the fight against CC.

In recent years, we have come to the realization that the immune microenforce impact tumor growth, metastasis, therapy, and prognosis (Hinshaw and Shevde, 2019; Lei et al., 2020). Studies have shown that next-generation sequencing has been found to be an innovative optional treatment for identifying actionable driver mutations and other markers (Menderes et al., 2016). We have found that lncRNA affects tumor development by influencing the immune response of tumors and infiltration of immune cells (Schwerdtfeger et al., 2021). It indicated that lncRNA, which is strongly relevant to the prognosis of patients, has been a significantly potential possible therapeutic drug for cancer and an innovative biotargeted molecule. In colorectal cancer, lncRNA SATB2-AS1 inhibits cancer metastasis through a regulation of SATB2, which impacts immune cells of the TIME (Xu et al., 2019).

Long noncoding RNA NEAT1 inhibits the progression of patients with early onset myocardial infarction by regulating the immune cell function (Gast et al., 2019). However, the method of effectively intervening to improve the prognosis of cervical cancer based on immune-related lncRNAs has not yet been fully clarified. It is in view of the vital role of immune infiltration in cancer development and proliferation that we have found new immune-related biomarkers for CC that may be useful for immunization therapy. In this study a solid CC immune-related risk signature was established using the profiles from TCGA TARGET GTEx dataset and TCGA dataset. Through multiple screenings of univariate Cox regression, LASSO regression, and multivariate Cox regression analysis, we identified six immune-related lncRNAs (AC006126.4, EGFR-AS1, RP4-647J21.1, LINC00925, EMX2OS, and BZRAP1-AS1) and also established a risk signature model that classified CC patients to low-risk and high-risk groups.

The prognostic value of immune-related lncRNAs in a variety of cancers has been confirmed by newly reported studies (Li et al., 2020a). In our study, we identified six lncRNAs as the hub signature. In the risk model, we found that AC006126.4 and EGFR-AS1 were risk-related genes, while RP4-647J21.1, LINC00925, EMX2OS, and BZRAP1-AS1 were protect genes. Some of these genes are reported to have taken part in the regulation of the immune response. lncRNA EGFR antisense RNA 1 (EGFR-AS1) is a transcription of 2.8-kb, transcribed on the antonymic chain of the epidermal growth factor receptor (EGFR), which is part of the family of receptor tyrosine kinases ErbB (Patel and Leung, 2012; Qi et al., 2016; Tan et al., 2017). It has been reported that the EGFR is essential in physiological processes just like cell migration, cell multiplication, and cell-cycle control (Wilusz et al., 2009) and that the overexpression of EGFR-AS1 is enough to cause resisting tyrosine kinase inhibitors and the downregulation of EGFR-AS1 which can induce the continuous tumor regression of squamous cell carcinoma (Tan et al., 2017). So far, there has been little research on the role of naturally occurring EGFR subtypes, and few studies have shown that other subtypes (such as secreted EGFR or sEGFR) are secreted in the plasma and may be prognostic for lung and cervical cancer (Halle et al., 2011; Lococo et al., 2015; Romero-Ventosa et al., 2015). In addition, the EGFR-AS1 mutation is thought to be associated with several cancers, including lung, stomach, kidney, and colorectal cancers (Hu et al., 2018; Wang et al., 2019b; Qi et al., 2019; Atef et al., 2021). In Wang’s study, EGFR-as1 may be involved in immune-related pathways to promote the progression of bladder cancer by upregulating the EGFR (Wang et al., 2020). Therefore, it is suggested that EGFR-AS1 may be a potential bridge between gynecology and immuno-oncology. LINC00925, also known as MIR9-3HG, has shown that it is strongly linked to the occurrence and development of cervical cancer in current studies (Wu et al., 2018; Gast et al., 2019). Chen’s study suggests that as an miRNA-host lncRNA, LINC00925 exhibited high expression in cervical cancer patients, prolonging their survival time (Chen et al., 2021); the aforementioned research results are consistent with ours, but the detailed mechanism of MIR9-3HG in CC development is not known. Studies have shown that removing MIR9-3HG inhibits the proliferation of cells and promotes apoptosis in CC (Li et al., 2021). MIR9‐3HG was reported as an immune‐related lnRrNA introduced in hepatocellular carcinoma (LIHC) and presented in all immune cell populations except NK CD56dim in patients (Li et al., 2020b). With regard to the immunological function of MIR9-3HG in a variety of immune cells, we believe that MIR9-3HG is a wide participation in tumor invasion and metastasis. Similar to LINC00925, EMX2OS is another key signature in CC that affects prognosis (Wu et al., 2018; Zheng et al., 2020). Recent research has shown EMX2OS as a novel lncRNA potential immune-related biomarker for acute rejection and graft loss of renal allograft (Zhang et al., 2020). In addition, EMX2OS was reported to play a role in myalgic encephalomyelitis/chronic fatigue syndrome (ME/CFS), leading to speculation that it is involved in immunological diseases or in stress responses (Yang et al., 2018). We have found that EMX2OS, which has been expressed in ovarian cancer, may inhibit tumor development and development by acting on PD-L1 (procedural cell death protein 1) through the EMX2OS/miR-654/AKT3 axis (Duan et al., 2020). BZRAP1-AS1 is a low expression in CC that has been found in accordance with our expectations (Zheng et al., 2020). However, BZRAP1-AS1, which is identified as highly expressed in hepatocellular carcinoma tissue and cells, prevents the proliferation, migration, and angiogenesis of human umbilical vein endothelial cell (HUVEC) in cancer cells (Wang et al., 2019c). As for the other two genes named AC006126.4 and RP4-647J21.1, which are located on human chromosome 19 and chromosome 7, respectively, there are relatively few studies on them to date. Although their role in CC is currently unclear, we can predict that they may be novel biomarkers associated with CC.

The view that the interaction between immune cells and cancer cells in tumor tissue reshapes the TIME and that the inflammatory microenvironment affects clinical therapeutic effect is more widely recognized (Liu et al., 2018).

From the previous studies, we have found that a reduction in the proportion of CD4 T cells in the CC microenvironment can lead to a reversal of CD4/CD8 ratios in tumor-infused lymphocytes (TILs) that inhibit anti-tumor immunological function (Sheu et al., 20011950; Shah et al., 2011). Another research shows that CC cells can tempt antigen-presenting cells (APCs) to form a broken immune environment that affects the survival and prognosis of patients (Cao et al., 2019). The aforementioned two points are consistent with the results of the xCell algorithm we studied. In addition, we analyzed 22 immune cell subtypes using the CIBERSORT algorithm to explore the most enrichment of infiltration cells are CD8^+^ T cells and macrophages in the immune microenvironment of CC. An earlier study suggested that the average quantity of CD8 T cells, a key cytotoxic lymphocyte that targeted cancer cells and monitored them immunologically, was more abundant in the CC tissue than in peripheral blood (Wu et al., 2020). Activated CD4^+^ T cells promote the activation of cytotoxic immune responses that require CD8^+^ T cells to function (Ostroumov et al., 2018). In the initial stage of immune function, CD8^+^ T cells were in a positive relationship with activated memory CD4^+^ T cells, while there was an inverse relationship with resting memory CD4^+^ T cells. Nonactivated M0 macrophages, pro-inflammatory M1 macrophages, and immunosuppressive M2 macrophages belong to the macrophage family (Chen et al., 2017; Ludtka et al., 2021). Previous studies have shown that M0, M1, and M2 macrophages are significantly enriched in CC, especially concentrating on M2, which goes along with our findings (Petrillo et al., 2015). Macrophages are a kind of immune innate cells that can participate in the intricate immunological procedure with strong plasticity and heterogeneity (Peet et al., 2020). The different levels of cell infiltration in CC indicate that the variation of immune-related signatures is a clear inherent characteristic that can be used to reflect personally, particularly a variation in the face of cancer attack, which has important clinical significance. Research has shown that it is effective in treating and preventing the development of various cancers by regulating inflammation (Wattenberg and Beatty, 2020). The significantly different immune cells we have analyzed may serve as inflammatory biomarkers which will be an important predictor of prognosis for CC patients.

In addition, the initiation and monitoring of protective immune cells are inhibited by immune checkpoints such as CTLA-4 and PD-1/PD-L1, which are a varistor of immune response regulation. So our research showed an increase in the expression of immune checkpoints in high-risk patients of the risk model, which impels us to weigh how important it is in the prediction of the immune checkpoint inhibitor (ICI) response. Through the TIDE algorithm, we have successfully demonstrated that the risk score of the immune-related risk model can effectively predict the immunotherapy response. As we concluded, immunotherapy responses were higher in the low-risk group (124/137) than in the high-risk group (94/137), indicating a significantly negative correlation between risk scores and immunotherapy response. All these confirm that immune-related gene signatures are an effective tumor biomarker for predicting response to immunotherapy. The Wilcoxon rank-sum test result exhibited that the high-risk group was more susceptive to whole compounds and displays the 12 compounds (bortezomib, docetaxel, doxorubicin, FTI.277, imatinib, metformin, mitomycin.C, MK.2206, MS.275, pazopanib, shikonin, and VX.680) that might be used for further analysis in patients with CC. The same was true for the difference analysis with IC50 of chemotherapeutics between the high- and low-risk groups. This study focuses on the immune-related lncRNAs, and there are some limitations to our study. The underlying mechanisms underlying the relationship between CC immune genes and survival outcomes need to be further analyzed by *in vivo* and *in vitro* assays. Their exploratively selected signatures and constructed immune-related risk model may provide clues for discovering the mechanisms and functions of immune genes for CC patients. In addition, we used a variety of methods to verify that this new model algorithm is optimum, and the subsequent analysis of this study adopted this model. Therefore, we are convinced that our conclusions were quite reliable.

Taken together, our study innovatively identified and validated six immune-related lncRNAs of CC and found an immune-related risk model for predicting clinical outcomes, indicated the immune cell infiltration intensity in the TIME, and predicted potential compounds in the immunotherapy treatment for CC. The key signatures we have selected may define a new treatment strategy that will be the new immune biomarkers for CC immunotherapy in the future.

## Data Availability

The datasets presented in this study can be found in online repositories. The names of the repository/repositories and accession number(s) can be found in the article/Supplementary Material.
