# Supplementary material for: Exploration of the Immune-Related Long Noncoding RNA Prognostic Signature and Inflammatory Microenvironment for Cervical Cancer
Source: Front Pharmacol. 2022 May 19;13:870221. doi: 10.3389/fphar.2022.870221 (PMC9161697; doi:10.3389/fphar.2022.870221)
Supplement: Supplementary file 1 [file Table1.docx]

Table S1.Characteristics of CC patients.

|  | level | High Risk | Low Risk | p-value |
| --- | --- | --- | --- | --- |
| n |  | 137 | 137 |  |
| Age (%) | <35 | 21 (15.3) | 21 (15.3) | 0.756 |
|  | >=65 | 18 (13.1) | 14 (10.2) |  |
|  | 35 to 65 | 93 (67.9) | 99 (72.3) |  |
|  | NA | 5 ( 3.6) | 3 ( 2.2) |  |
| Stage (%) | 0 | 3 ( 2.2) | 3 ( 2.2) | 0.142 |
|  | 1 | 63 (46.0) | 80 (58.4) |  |
|  | 2 | 28 (20.4) | 31 (22.6) |  |
|  | 3 | 24 (17.5) | 13 ( 9.5) |  |
|  | 4 | 14 (10.2) | 7 ( 5.1) |  |
|  | NA | 5 ( 3.6) | 3 ( 2.2) |  |
| Grade (%) | 0 | 18 (13.1) | 8 ( 5.8) | 0.047 |
|  | 1 | 4 ( 2.9) | 11 ( 8.0) |  |
|  | 2 | 53 (38.7) | 67 (48.9) |  |
|  | 3 | 57 (41.6) | 47 (34.3) |  |
|  | 4 | 0 ( 0.0) | 1 ( 0.7) |  |
|  | NA | 5 ( 3.6) | 3 ( 2.2) |  |
| Pathologic_M (%) | 0 | 124 (90.5) | 131 (95.6) | 0.227 |
|  | 1 | 8 ( 5.8) | 3 ( 2.2) |  |
|  | NA | 5 ( 3.6) | 3 ( 2.2) |  |
| Pathologic_N (%) | 0 | 107 (78.1) | 106 (77.4) | 0.714 |
|  | 1 | 25 (18.2) | 28 (20.4) |  |
|  | NA | 5 ( 3.6) | 3 ( 2.2) |  |
| Pathologic_T (%) | 0 | 30 (21.9) | 19 (13.9) | <0.001 |
|  | 1 | 51 (37.2) | 74 (54.0) |  |
|  | 2 | 27 (19.7) | 38 (27.7) |  |
|  | 3 | 15 (10.9) | 2 ( 1.5) |  |
|  | 4 | 9 ( 6.6) | 1 ( 0.7) |  |
|  | NA | 5 ( 3.6) | 3 ( 2.2) |  |
| Smoking (%) | NA | 5 ( 3.6) | 3 ( 2.2) | 0.084 |
|  | No Smoking | 18 (13.1) | 8 ( 5.8) |  |
|  | Smoking | 114 (83.2) | 126 (92.0) |  |
| BMI (%) | Low Weight | 7 ( 5.1) | 4 ( 2.9) | 0.106 |
|  | NA | 26 (19.0) | 20 (14.6) |  |
|  | Normal | 37 (27.0) | 26 (19.0) |  |
|  | Over Weight | 67 (48.9) | 87 (63.5) |  |
